# 4,6-Dimeth­oxy-2-(methyl­sulfan­yl)pyrimidinium chloride

**DOI:** 10.1107/S1600536809055779

**Published:** 2010-01-09

**Authors:** Madhukar Hemamalini, Hoong-Kun Fun

**Affiliations:** a X-ray Crystallography Unit, School of Physics, Universiti Sains Malaysia, 11800 USM, Penang, Malaysia

## Abstract

In the title compound, C_7_H_11_N_2_O_2_S^+^·Cl^−^, the 4,6-dimeth­oxy-2-(methyl­sulfan­yl)pyrimidinium cation is essentially planar (r.m.s. deviation = 0.043 Å). In the crystal, the anions and cations are connected by inter­molecular N—H⋯Cl and C—H⋯Cl hydrogen bonds, forming a two-dimensional network parallel to (011). Adjacent networks are cross-linked *via* π–π inter­actions involving the pyrimidinium ring [centroid–centroid distance = 3.5501 (8) Å].

## Related literature

For general background to substituted pyrimidines, see: Salas *et al.* (1995 ▶); Holy *et al.* (1974 ▶); Hunt *et al.* (1980 ▶); Baker & Santi (1965 ▶); Balasubramani & Fun (2009 ▶); For bond-length data, see: Allen *et al.* (1987 ▶). For the stability of the temperature controller used for the data collection, see: Cosier & Glazer (1986 ▶).
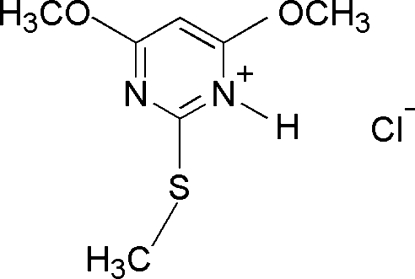


## Experimental

### 

#### Crystal data

C_7_H_11_N_2_O_2_S^+^·Cl^−^
*M* *_r_* = 222.69
Triclinic, 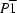

*a* = 6.6934 (2) Å
*b* = 8.4713 (2) Å
*c* = 8.8123 (2) Å
α = 79.774 (1)°
β = 87.294 (1)°
γ = 84.494 (1)°
*V* = 489.24 (2) Å^3^
*Z* = 2
Mo *K*α radiation
μ = 0.57 mm^−1^
*T* = 100 K
0.32 × 0.22 × 0.14 mm

#### Data collection

Bruker SMART APEXII CCD area-detector diffractometer
Absorption correction: multi-scan (*SADABS*; Bruker, 2009 ▶) *T*_min_ = 0.836, *T*_max_ = 0.922
9438 measured reflections
2126 independent reflections
1889 reflections with *I* > 2σ(*I*)
*R*_int_ = 0.022

#### Refinement

*R*[*F* ^2^ > 2σ(*F* ^2^)] = 0.027
*wR*(*F* ^2^) = 0.078
*S* = 1.03
2126 reflections
125 parameters
H atoms treated by a mixture of independent and constrained refinement
Δρ_max_ = 0.41 e Å^−3^
Δρ_min_ = −0.31 e Å^−3^

### 

Data collection: *APEX2* (Bruker, 2009 ▶); cell refinement: *SAINT* (Bruker, 2009 ▶); data reduction: *SAINT*; program(s) used to solve structure: *SHELXTL* (Sheldrick, 2008 ▶); program(s) used to refine structure: *SHELXTL*; molecular graphics: *SHELXTL*; software used to prepare material for publication: *SHELXTL* and *PLATON* (Spek, 2009 ▶).

## Supplementary Material

Crystal structure: contains datablocks global, I. DOI: 10.1107/S1600536809055779/ci5011sup1.cif

Structure factors: contains datablocks I. DOI: 10.1107/S1600536809055779/ci5011Isup2.hkl

Additional supplementary materials:  crystallographic information; 3D view; checkCIF report

## Figures and Tables

**Table 1:** Hydrogen-bond geometry (Å, °)

*D*—H⋯*A*	*D*—H	H⋯*A*	*D*⋯*A*	*D*—H⋯*A*
N2—H2⋯Cl1^i^	0.96 (3)	2.00 (3)	2.9606 (13)	172 (2)
C6—H6*A*⋯Cl1^ii^	0.96	2.77	3.4896 (16)	132
C6—H6*B*⋯Cl1	0.96	2.80	3.7002 (15)	157
C7—H7*A*⋯Cl1^iii^	0.96	2.76	3.5524 (15)	141

Symmetry codes: (i) 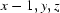; (ii) 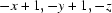; (iii) 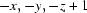.

**Fig. 1.**
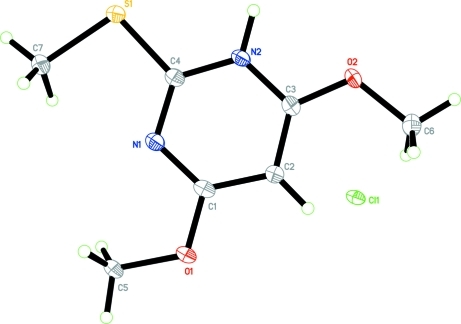
The molecular structure of the title compound, showing 50% probability displacement ellipsoids and the atom-numbering scheme.

**Fig. 2.**
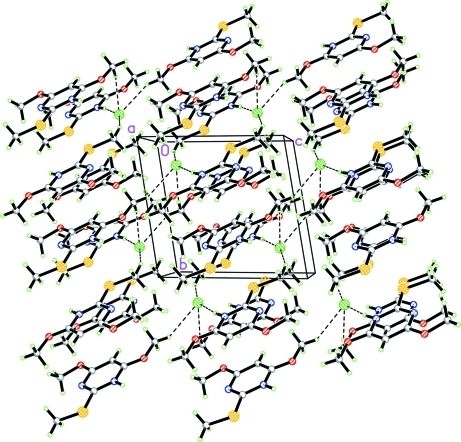
The crystal packing of the title compound, viewed along the a axis.

## supplementary crystallographic information

### Comment

Pyrimidine and aminopyrimidine derivatives are biologically important compounds as they occur in nature as components of nucleic acids. Some aminopyrimidine derivatives are used as antifolate drugs (Hunt *et al.* 1980; Baker & Santi, 1965). We have recently reported the crystal structure of 4,6-dimethoxy-2(methylsulfanyl)pyrimidine (Balasubramani & Fun, 2009). In continuation of our studies of pyrimidinium derivatives, the crystal structure determination of the title compound has been undertaken.

The asymmetric unit of the title compound (Fig. 1) consists of a chloride anion and a 4,6-dimethoxy-2(methylsulfanyl)pyridinium cation. Protonation of the pyrimidine base on the N2 site is reflected in a change in the bond angle. The C4—N1—C1 angle at unprotonated atom N1 is 116.84(13 Å, whereas for protonated atom N2 the C4—N2—C3 angle is 120.03 (13) Å. The bond lengths and angles are normal (Allen *et al.* 1987).

In the crystal packing (Fig. 2), atoms N2, C7 and C6 act as donors for intermolecular N—H···Cl and C—H···Cl hydrogen bonds with symmetry related chloride anions (Table 1), forming a two-dimensional network parallel to the (011). Adjacent networks are cross-linked *via*π–π interactions involving the pyrimidinium ring with centroid···centroid  distance = 3.5501 (8) Å (symmetry code  -x, 1-y, 1-z).

### Experimental

To a hot methanol solution (20 ml) of 4,6-dimethoxy-2-(methylsulfanyl)pyrimidine (46 mg, Aldrich) was added a few drops of hydrochloric acid. The solution was warmed over a water bath for a few minutes. The resulting solution was allowed to cool slowly to room temperature. Crystals of the title compound appeared from the mother liquor after a few days.

### Refinement

Atom H2 was located in a difference Fourier map and refined freely. The remaining H atoms were positioned geometrically [C–H = 0.93 or 0.96 Å] and were refined using a riding model, with *U*_iso_(H) = 1.2 or 1.5 *U*_eq_(C). A rotating group model was applied to the methyl groups.

### Crystal data

**Table:**

C_7_H_11_N_2_O_2_S^+^·Cl^−^	*Z* = 2
*M**_r_* = 222.69	*F*(000) = 232
Triclinic, *P*1	*D*_x_ = 1.512 Mg m^−^^3^
Hall symbol: -P 1	Mo *K*α radiation, λ = 0.71073 Å
*a* = 6.6934 (2) Å	Cell parameters from 6382 reflections
*b* = 8.4713 (2) Å	θ = 2.4–30.1°
*c* = 8.8123 (2) Å	µ = 0.57 mm^−^^1^
α = 79.774 (1)°	*T* = 100 K
β = 87.294 (1)°	Block, colourless
γ = 84.494 (1)°	0.32 × 0.22 × 0.14 mm
*V* = 489.24 (2) Å^3^	

### Data collection

**Table:**

Bruker SMART APEXII CCD area-detector diffractometer	2126 independent reflections
Radiation source: fine-focus sealed tube	1889 reflections with *I* > 2σ(*I*)
graphite	*R*_int_ = 0.022
φ and ω scans	θ_max_ = 27.0°, θ_min_ = 2.4°
Absorption correction: multi-scan (*SADABS*; Bruker, 2009)	*h* = −7→8
*T*_min_ = 0.836, *T*_max_ = 0.922	*k* = −9→10
9438 measured reflections	*l* = −11→11

### Refinement

**Table:**

Refinement on *F*^2^	Primary atom site location: structure-invariant direct methods
Least-squares matrix: full	Secondary atom site location: difference Fourier map
*R*[*F*^2^ > 2σ(*F*^2^)] = 0.027	Hydrogen site location: inferred from neighbouring sites
*wR*(*F*^2^) = 0.078	H atoms treated by a mixture of independent and constrained refinement
*S* = 1.02	*w* = 1/[σ^2^(*F*_o_^2^) + (0.0453*P*)^2^ + 0.2271*P*] where *P* = (*F*_o_^2^ + 2*F*_c_^2^)/3
2126 reflections	(Δ/σ)_max_ = 0.001
125 parameters	Δρ_max_ = 0.41 e Å^−^^3^
0 restraints	Δρ_min_ = −0.31 e Å^−^^3^

### Special details

**Table:**

Experimental. The crystal was placed in the cold stream of an Oxford Cyrosystems Cobra open-flow nitrogen cryostat (Cosier & Glazer, 1986) operating at 100.0 (1) K.
Geometry. All e.s.d.'s (except the e.s.d. in the dihedral angle between two l.s. planes) are estimated using the full covariance matrix. The cell e.s.d.'s are taken into account individually in the estimation of e.s.d.'s in distances, angles and torsion angles; correlations between e.s.d.'s in cell parameters are only used when they are defined by crystal symmetry. An approximate (isotropic) treatment of cell e.s.d.'s is used for estimating e.s.d.'s involving l.s. planes.
Refinement. Refinement of *F*^2^ against ALL reflections. The weighted *R*-factor *wR* and goodness of fit *S* are based on *F*^2^, conventional *R*-factors *R* are based on *F*, with *F* set to zero for negative *F*^2^. The threshold expression of *F*^2^ > σ(*F*^2^) is used only for calculating *R*-factors(gt) *etc*. and is not relevant to the choice of reflections for refinement. *R*-factors based on *F*^2^ are statistically about twice as large as those based on *F*, and *R*- factors based on ALL data will be even larger.

### Fractional atomic coordinates and isotropic or equivalent isotropic displacement parameters (Å^2^)

**Table:**

	*x*	*y*	*z*	*U*_iso_*/*U*_eq_	
S1	−0.25972 (5)	0.07775 (4)	0.60806 (4)	0.01603 (11)	
O1	0.34406 (15)	0.34047 (13)	0.69667 (11)	0.0171 (2)	
O2	0.07854 (16)	0.42350 (13)	0.19722 (11)	0.0174 (2)	
N1	0.06343 (18)	0.22322 (15)	0.65196 (14)	0.0143 (3)	
N2	−0.05961 (19)	0.27213 (15)	0.40095 (14)	0.0145 (3)	
C1	0.2115 (2)	0.31501 (18)	0.59794 (17)	0.0146 (3)	
C2	0.2360 (2)	0.38881 (18)	0.44398 (17)	0.0154 (3)	
H2A	0.3426	0.4501	0.4099	0.018*	
C3	0.0918 (2)	0.36459 (17)	0.34645 (16)	0.0144 (3)	
C4	−0.0672 (2)	0.20134 (17)	0.55095 (16)	0.0140 (3)	
C5	0.3256 (2)	0.2515 (2)	0.85378 (17)	0.0187 (3)	
H5A	0.4342	0.2717	0.9131	0.028*	
H5B	0.3305	0.1384	0.8513	0.028*	
H5C	0.2001	0.2860	0.9002	0.028*	
C6	0.2314 (2)	0.52752 (19)	0.12795 (18)	0.0192 (3)	
H6A	0.2041	0.5656	0.0211	0.029*	
H6B	0.3610	0.4679	0.1365	0.029*	
H6C	0.2299	0.6176	0.1806	0.029*	
C7	−0.1949 (2)	0.0026 (2)	0.80670 (17)	0.0189 (3)	
H7A	−0.2778	−0.0817	0.8498	0.028*	
H7B	−0.2161	0.0886	0.8650	0.028*	
H7C	−0.0563	−0.0391	0.8106	0.028*	
Cl1	0.63752 (5)	0.19691 (4)	0.19642 (4)	0.01942 (12)	
H2	−0.160 (4)	0.258 (3)	0.331 (3)	0.047 (6)*	

### Atomic displacement parameters (Å^2^)

**Table:**

	*U*^11^	*U*^22^	*U*^33^	*U*^12^	*U*^13^	*U*^23^
S1	0.0151 (2)	0.0185 (2)	0.01466 (19)	−0.00449 (14)	−0.00315 (13)	−0.00105 (14)
O1	0.0169 (5)	0.0222 (6)	0.0127 (5)	−0.0044 (4)	−0.0054 (4)	−0.0020 (4)
O2	0.0207 (6)	0.0205 (6)	0.0106 (5)	−0.0057 (4)	−0.0044 (4)	0.0016 (4)
N1	0.0147 (6)	0.0160 (6)	0.0125 (6)	−0.0012 (5)	−0.0023 (5)	−0.0030 (5)
N2	0.0153 (6)	0.0164 (6)	0.0123 (6)	−0.0027 (5)	−0.0045 (5)	−0.0018 (5)
C1	0.0148 (7)	0.0156 (7)	0.0141 (7)	0.0013 (6)	−0.0044 (5)	−0.0048 (5)
C2	0.0155 (7)	0.0171 (7)	0.0136 (7)	−0.0037 (6)	−0.0020 (5)	−0.0016 (6)
C3	0.0166 (7)	0.0140 (7)	0.0123 (7)	0.0000 (6)	−0.0018 (5)	−0.0019 (5)
C4	0.0139 (7)	0.0144 (7)	0.0136 (7)	0.0007 (5)	−0.0026 (5)	−0.0029 (5)
C5	0.0201 (8)	0.0244 (8)	0.0115 (7)	−0.0044 (6)	−0.0058 (6)	−0.0001 (6)
C6	0.0225 (8)	0.0189 (8)	0.0157 (7)	−0.0048 (6)	−0.0003 (6)	0.0002 (6)
C7	0.0208 (8)	0.0216 (8)	0.0137 (7)	−0.0049 (6)	−0.0026 (6)	0.0007 (6)
Cl1	0.0191 (2)	0.0229 (2)	0.01680 (19)	−0.00531 (15)	−0.00808 (14)	−0.00133 (14)

### Geometric parameters (Å, °)

**Table:**

S1—C4	1.7380 (16)	C2—C3	1.375 (2)
S1—C7	1.8113 (15)	C2—H2A	0.93
O1—C1	1.3292 (17)	C5—H5A	0.96
O1—C5	1.4598 (18)	C5—H5B	0.96
O2—C3	1.3251 (17)	C5—H5C	0.96
O2—C6	1.4556 (19)	C6—H6A	0.96
N1—C4	1.3244 (18)	C6—H6B	0.96
N1—C1	1.336 (2)	C6—H6C	0.96
N2—C4	1.3519 (19)	C7—H7A	0.96
N2—C3	1.358 (2)	C7—H7B	0.96
N2—H2	0.97 (3)	C7—H7C	0.96
C1—C2	1.399 (2)		
			
C4—S1—C7	99.97 (7)	O1—C5—H5A	109.5
C1—O1—C5	116.16 (12)	O1—C5—H5B	109.5
C3—O2—C6	116.90 (12)	H5A—C5—H5B	109.5
C4—N1—C1	116.84 (13)	O1—C5—H5C	109.5
C4—N2—C3	120.03 (13)	H5A—C5—H5C	109.5
C4—N2—H2	121.1 (14)	H5B—C5—H5C	109.5
C3—N2—H2	118.8 (14)	O2—C6—H6A	109.5
O1—C1—N1	118.30 (13)	O2—C6—H6B	109.5
O1—C1—C2	117.18 (13)	H6A—C6—H6B	109.5
N1—C1—C2	124.51 (13)	O2—C6—H6C	109.5
C3—C2—C1	115.49 (14)	H6A—C6—H6C	109.5
C3—C2—H2A	122.3	H6B—C6—H6C	109.5
C1—C2—H2A	122.3	S1—C7—H7A	109.5
O2—C3—N2	112.49 (12)	S1—C7—H7B	109.5
O2—C3—C2	127.29 (14)	H7A—C7—H7B	109.5
N2—C3—C2	120.22 (13)	S1—C7—H7C	109.5
N1—C4—N2	122.85 (14)	H7A—C7—H7C	109.5
N1—C4—S1	120.43 (11)	H7B—C7—H7C	109.5
N2—C4—S1	116.73 (11)		
			
C5—O1—C1—N1	5.85 (19)	C4—N2—C3—C2	−1.1 (2)
C5—O1—C1—C2	−175.01 (13)	C1—C2—C3—O2	178.56 (14)
C4—N1—C1—O1	179.41 (12)	C1—C2—C3—N2	−1.0 (2)
C4—N1—C1—C2	0.3 (2)	C1—N1—C4—N2	−2.6 (2)
O1—C1—C2—C3	−177.67 (13)	C1—N1—C4—S1	177.53 (10)
N1—C1—C2—C3	1.4 (2)	C3—N2—C4—N1	3.0 (2)
C6—O2—C3—N2	178.64 (12)	C3—N2—C4—S1	−177.09 (10)
C6—O2—C3—C2	−0.9 (2)	C7—S1—C4—N1	−4.31 (13)
C4—N2—C3—O2	179.30 (12)	C7—S1—C4—N2	175.82 (11)

### Hydrogen-bond geometry (Å, °)

**Table:**

*D*—H···*A*	*D*—H	H···*A*	*D*···*A*	*D*—H···*A*
N2—H2···Cl1^i^	0.96 (3)	2.00 (3)	2.9606 (13)	172 (2)
C6—H6A···Cl1^ii^	0.96	2.77	3.4896 (16)	132
C6—H6B···Cl1	0.96	2.80	3.7002 (15)	157
C7—H7A···Cl1^iii^	0.96	2.76	3.5524 (15)	141

Symmetry codes: (i) *x*−1, *y*, *z*; (ii) −*x*+1, −*y*+1, −*z*; (iii) −*x*, −*y*, −*z*+1.
